# Cryptococcosis due to *Cryptococcus gattii* VGII in southeast Brazil: The One Health approach revealing a possible role for domestic cats

**DOI:** 10.1016/j.mmcr.2019.04.004

**Published:** 2019-04-17

**Authors:** Fábio Brito-Santos, Rosani Santos Reis, Rowena Alves Coelho, Rodrigo Almeida-Paes, Sandro Antônio Pereira, Luciana Trilles, Wieland Meyer, Bodo Wanke, Márcia dos Santos Lazéra, Isabella Dib Ferreira Gremião

**Affiliations:** a Mycology Laboratory, National Institute of Infectious Diseases Evandro Chagas (INI), Fiocruz, Rio de Janeiro, Brazil; b Laboratory of Clinical Research on Dermatozoonosis in Domestic Animals, National Institute of Infectious Diseases Evandro Chagas (INI), FIOCRUZ, Rio de Janeiro, Brazil; c Molecular Mycology Research Laboratory, Westmead Institute for Medical Research, Sydney, NSW, Australia

**Keywords:** *Cryptococcus gattii*, VGII, MLST, Cryptococcosis, Cat

## Abstract

Two cats infected by *C. gattii,* presented lesions on the nasal region and respiratory signs. Strains were typed as molecular type VGII, mating type alpha, MLST subtypes ST442 and ST185. Since Rio de Janeiro is known as an endemic area for *C. neoformans* VNI, these cases might be a warning for a possible emergence of *C. gattii* VGII in southeast Brazil.

## 1 Introduction

Cryptococcosis is a worldwide-distributed fungal disease of humans and animals caused by members of the *Cryptococcus neoformans* and *Cryptococcus gattii* complexes [1]. Both species have been reported to infect domestic pets, livestock, as well as birds and aquatic mammals [2]. Since 1999, the *C. gattii* molecular type VGII, has emerged as an important pathogen of humans and animals as result of the first multi-species outbreak of cryptococcosis in British Columbia, Canada [3]. In the North (N) and Northeast (NE) regions of Brazil, *C. gattii*, mainly the molecular type VGII, prevails as the cause of cryptococcal meningitis in children and young adults, as well as in several environments, like domiciliary dust, soil, and trees. However, in the southern regions of the country, *C. neoformans* predominates, and infections by *C. gattii* VGII are rare and usually considered as imported cases [4].

Cryptococcosis is the most common systemic mycosis in cats. These animals were found to be particularly more susceptible to infections by *C. neoformans* than dogs in studies conducted in Western Australia [5]. Feline cryptococcosis is frequently reported in Australia, Canada and the United States [6].

In Brazil, very few animal cases of cryptococcosis have been documented [7,8] and most of them do not discriminate the causative species complexes: *C. neoformans* from *C. gattii*. We describe herein two cases of feline cryptococcosis due to *C. gattii* in the state of Rio de Janeiro, southeast Brazil, focusing on the differential diagnosis, treatment and clinical outcome, along with the phenotypic identification, molecular typing and *in vitro* antifungal susceptibility of the agents. Moreover, a role for these animals in cryptococcosis within the concept of One Health is discussed.

## 2 Case presentations

**Fig. 1:**
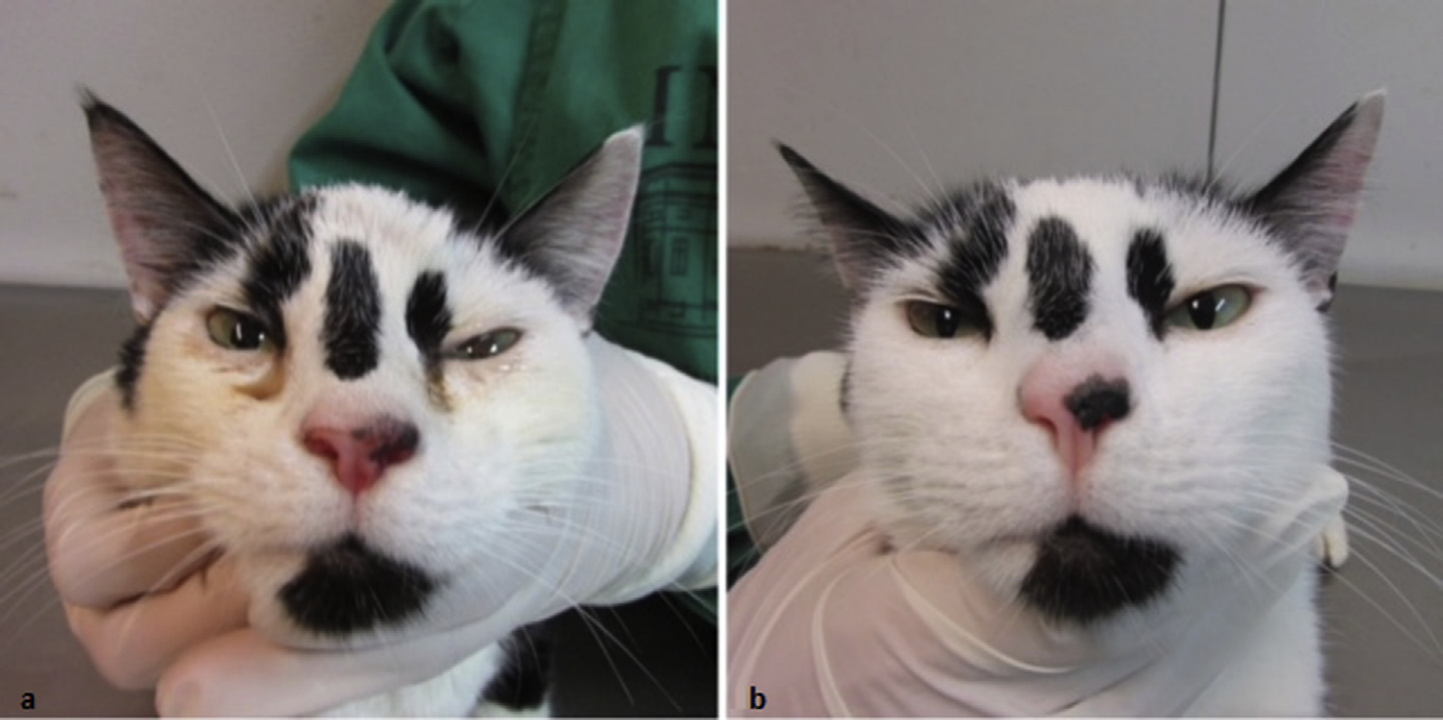
Case 1. A) Cat presenting a firm subcutaneous swelling over the forehead, bilateral ocular seropurulent secretion, and polyp-like mass in the nostrils. B) Case 1 after the disappearance of all the clinical signs initially presented, which occurred after six months of fluconazole therapy.

Two cats with suspicion of cryptococcosis based on clinical signs and positive cytopathological examination, were referred to a reference center for fungal diseases in animals in Rio de Janeiro, southeast Brazil. The cat from case 1 was a 4-year-old female cross-breed cat, castrated, weighing 3.3 kg. At the clinical examination, this cat presented a firm subcutaneous swelling over the forehead, bilateral ocular seropurulent secretion, and polyp-like mass in the nostrils. Dyspnea and nasal discharge were also observed (Fig. 1a). Exudate from the lesion located on the forehead obtained by aspiration was collected for cytopathological examination. Yeasts suggestive of *Cryptococcus* spp. were observed in Panotic-stained smears. Exudate from the same lesion and secretion from the nasal cavities obtained with a swab were seeded on to Sabouraud-dextrose agar. The physiologic characterization was performed as follows: the isolated yeasts were tested for thermotolerance at 37 °C, melanin production on Niger seed agar, and assimilation of carbon and nitrogen compounds. The species complexes *C. neoformans* and *C. gattii* were differentiated via incubation on canavanine-glycine-bromothymol blue (CGB) medium. The mating type was determined applying polymerase chain reaction using specific primers for the pheromone genes. Subtyping was performed according to the ISHAM consensus multi-locus sequence typing scheme for *C. neoformans* and *C. gattii* [9]. The sequences were manually edited using the software Sequencer 4.10.1, and the allele types (AT) and the sequence types (ST) were identified via the MLST webpage (http://mlst.mycologylab.org/). The results revealed the presence of *C. gattii* VGII, mat *alfa*, sequence type ST442. Antifungal susceptibility testing was performed according to the CLSI M27-A3 guideline. The drugs tested were fluconazole, voriconazole, itraconazole, flucytosine, and amphotericin B. The minimal inhibitory concentrations (MICs) were evaluated by means of the epidemiologic cutoff values (ECVs) suggested for the specific molecular types of *C. gattii* [10,11]. These ECVs allow the classification of strains as Wild Type (WT) or Non-Wild Type (Non-WT). The MICs in μg/ml were as follows: 4.0 for fluconazole, 0.12 for voriconazole, 2.0 for flucytosine, and 0.12 for amphotericin B, all of them were consistent with the WT population. MIC of 1 μg · ml^−1^ for itraconazole was consistent with the non-WT population (Table 1).

**Table 1:** Laboratory results of the two cryptococcosis feline cases herein reported.

Case report	Molecular ID	Mating type	MLST (sequence type)	AST (CLSI M27-A3)		
				Antifungal	MIC	Classification
Case 1	*C. gattii* VGII	*alfa*	ST442	Fluconazole	4.0	aWT
				Voriconazole	0.12	WT
				Itraconazole	1.0	bNon-WT
				Amphotericin	0.12	WT
				Flucytosine	2.0	WT
Case 2	*C. gattii* VGII	*alfa*	ST185	Fluconazole	16	WT
				Voriconazole	1.0	Non-WT
				Itraconazole	1.0	Non-WT
				Amphotericin	0.12	WT
				Flucytosine	4.0	WT

a Wild Type (WT).

b Non-Wild Type (Non-WT).

Fluconazole (100 mg PO) was prescribed once a day (day 0), and the cat was followed up every 30 days for clinical evaluation. After the disappearance of all the clinical signs initially presented, which occurred after six months of therapy, the cat was discharged (Fig. 1b).

**Fig. 2:**
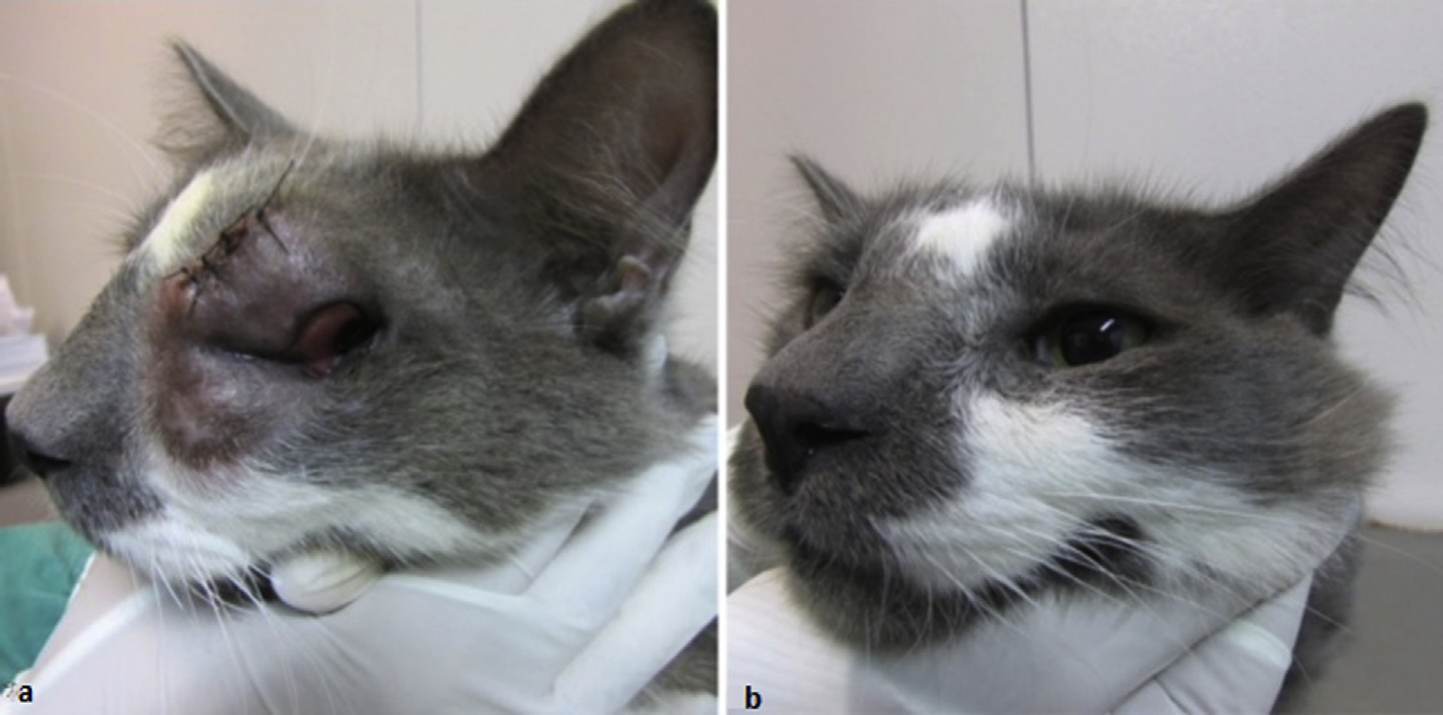
Case 2. A) Soft subcutaneous swelling over the left periocular medial region, a lesion on the conjunctiva of the left eye and polyp-like mass in the nostrils. B) Cat presenting clinical cure occurred after five months of fluconazole therapy.

The cat from case 2 was a 6-year-old male cross-breed cat, castrated, positive for feline leukaemia virus (FeLV), weighing 5.2 kg. The cat had been previously treated at a private veterinary clinic with itraconazole (100 mg PO, q24h) for one month, with no clinical response. At clinical examination, a soft subcutaneous swelling over the left periocular medial region, a lesion on the conjunctiva of the left eye, polyp-like mass in the nostrils and sneezing were observed (Fig. 2a). Exudate from the conjunctival lesion and nasal secretion samples were seeded on to Sabouraud-dextrose agar. The physiologic characterization was performed and revealed the presence of *C. gattii.* The isolates were further characterized as VGII, mat *alfa*, sequence type ST185, which were identified according to the techniques described for the first case (see above). The antifungal susceptibility testing and the ECVs classification of the strain were performed as described for case 1 (see above). The MIC's (μg/ml) were as follows: 16 for fluconazole, 4.0 for flucytosine, and 0.12 for amphotericin B, values consistent with WT population, as well as 1.0 for both itraconazole and voriconazole, which were consistent with the non-WT population (Table 1).

Itraconazole was replaced by fluconazole (100 mg PO, q24h) (day 0). The cat was followed up every 30 days for clinical evaluation. The clinical cure occurred after five months of therapy, when the cat was discharged (Fig. 2b).

## 3 Discussion

Since the first description in Brazil in 1971, only 27 other cases of feline cryptococcosis have been reported (Supplementary Table 1). Of these, *C. gattii* was properly identified in three cases, with one case due to the molecular type VGII. Thus, animal cryptococcosis is probably underdiagnosed and under-reported in Brazil, with the real incidence being severely biased. Herein, we report two cases of feline cryptococcosis due to *C. gattii* VGII in an urban area of the Southeast Brazil, largely known to be endemic for human cryptococcosis caused by *C. neoformans* VNI [4].

Environmental studies in Rio de Janeiro have isolated mainly *C. neoformans* VNI and *C. gattii* VGI, while *C. gattii* VGII has rarely been isolated [12]. Authors described the only Brazilian case of cryptococcosis in a cat whose isolate was assessed for its molecular type, and *C. gattii* VGII was identified [7]. Considering that cryptococcal infection in animals, reflects infections in human host from the same geographic area, studies about animal cryptococcosis in Rio de Janeiro reiterate the value of sentinel animal surveillance for this emerging infectious disease [13], reinforcing the need for a correct molecular identification of the agents.

Cats with localized cutaneous lesions can be treated successfully with fluconazole, which is the drug of choice for feline cryptococcosis. However, many cats with *C. gattii* VGII and VGIII infections in North America fail to respond to this azole, presenting persistent high antigen titers and frequent relapses. In these cases, itraconazole is a better option [5]. In previous reports from Brazil due to *C. gattii*, a cat presenting a nasal granuloma achieved clinical cure after six months therapy of itraconazole/5-flucytosine combination [7] and the other case, which presented a mass on the nasal region and partial obstruction of the nostrils, died 40 days after itraconazole therapy [8]. Case 2 failed to initially respond to itraconazole and the correspondent strain was a non-WT to itraconazole and voriconazole, with a non-WT organism showing reduced susceptibility to the agent being evaluated when compared to the WT population [11], as was observed in case 2. However, cases herein described were caused by strains classified as WT to fluconazole. Treatment with this drug lead to cure of both cases and no recurrence signs were observed over the following six months. Hence, more epidemiological studies are necessary to evaluate the trends in cryptococcal antifungal susceptibility to drugs and response to treatment, spread and circulation of subtypes in certain areas.

Cryptococcosis and sporotrichosis infections are thought to be acquired from the environment. However, in the Rio de Janeiro sporotrichosis epizootic, feline cases are mainly related to be acquired by scratches or bites from other infected cats [14], whereas cryptococcosis cases are thought to be acquired through inhalation of yeasts or basidiospores from the environment, with no individual to individual transmission ever been described [15]. Heteroresistance to itraconazole is intrinsic and usually increases the virulence of *C. gattii* [16]. This phenomenon may represent an additional mechanism that contributes to relapses of cryptococcosis in animals during itraconazole therapy. Itraconazole is the first choice therapy for sporotrichosis, since fluconazole is not effective *in vitro* against *Sporothrix* spp [17]. These differences in therapy of cryptococcosis and sporotrichosis reinforces the importance of differential diagnosis between these important infections.

The emergence of *C. gattii* in urban environments is a challenge because it comes with a great potential to generate outbreaks of cryptococcosis among immunocompetent individuals and animals. Future studies on the etiological agents of cryptococcosis in domestic animals in urban environments are necessary to understand the dynamics of this mycosis in these animals.

## Financial interest's declaration

The authors declare that no conflict of interests exist.

## Ethical approval

The procedures were approved by the Animal Ethics Committee under the license number LW 37/12.
