# Supplementary material for: Cryptococcosis due to Cryptococcus gattii VGII in southeast Brazil: The One Health approach revealing a possible role for domestic cats
Source: Med Mycol Case Rep. 2019 Apr 17;24:61–4. doi: 10.1016/j.mmcr.2019.04.004 (PMC6487353; doi:10.1016/j.mmcr.2019.04.004)
Supplement: Multimedia component 1 [file mmc1.doc]

**Supplementary Table 1**. Feline cryptococcosis cases in Brazil described between 1971-2019.

| **Year of publication** | **Reference** | **State** | **Number of cases** | **Species ID** | **Clinical Signs** |
| --- | --- | --- | --- | --- | --- |
| 1971 | Cruz *et al*.,[1] | Rio de Janeiro | 1 | *C. neoformans* | Swelling over the frontal region. |
| 2002 | Mendonça *et al.,*[2] | Minas Gerais | 1 | *C. neoformans* | Vegetative lesion with spongeous aspect in the right mandibula. |
| 2004 | Chiesa *et al*.,[3] | São Paulo | 18 | *C. neoformans* | No description of the clinical presentation. |
| 2006 | Juliano *et al*.,[4] | Mato Grosso do Sul | 1 | *C. neoformans* | Respiratory signs and swelling over the bridge of the nose. |
| 2011 | Martins *et al*.,[5] | Rio Grande do Sul | 1 | *C. neoformans* | Disseminated infection: multiple cutaneous nodules, anorexia, and apathy. |
| 2013 | Cardoso *et al*.,[6] | São Paulo | 1 | *C. gattii* | Nasal granuloma. |
| 2014 | De Paula *et al*.,[7] | Mato Grosso | 2 | *C. gattii* | Case 1. Respiratory distress, apathy, anorexia, cough and sneeze. Case 2. Ulcerated mass in nasal region, secretion and partial obstruction of nose. |
| 2018 | Lima *et al*.,[8] | São Paulo | 1 | *C. neoformans* | Respiratory signs and swelling over the bridge of the nose. |
| 2018 | Balda *et al*.,[9] | São Paulo | 1 | *C. neoformans* | Skin lesions on the nasal planum and the second digit of the left thoracic limb. |
| 2019 | Present study | Rio de Janeiro | 2 | *C. gattii* | Case1. Swelling over the forehead, polyp-like mass in the nostrils, bilateral ocular secretion and respiratory signs.  Case 2. Swelling over the left periocular medial region, polyp-like mass in the nostrils, sneezing. |

**References:**

[1] L.C.H. da Cruz, W.A. Chagas, J.B. de Figueiredo, Cryptococcosis in a cat. First case in Brazil, Cryptococcosis Cat First Case Braz. 1 (1971) 25–28.

[2] C. Mendonça, K. Waldemarin, H. Coelho, M. Lacerda, Criptococose na cavidade oral de um gato doméstico - relato de caso., Criptococose Na Cavidade Oral Um Gato Doméstico - Relato Caso. 5 (2002) 257–263.

[3] S. Chiesa, R. Castro, M. Otsuka, N. Michalany, C. Larsson Jr, C. Larsson, Cryptococcosis in São Paulo (Brazil): clinical and epidemiological features (1992-2003), Cryptococcosis São Paulo Braz. Clin. Epidemiol. Featur. 1992-2003. 15 (2004) 46.

[4] R.S. Juliano, A. Souza, R. Scheide, Criptococose felina, Criptococose Felina. 35 (2006) 65–70.

[5] D.B. Martins, R.A. Zanette, R.T. França, F. Howes, M.I. Azevedo, S.A. Botton, C. Mazzanti, S.T.A. Lopes, J.M. Santurio, Massive cryptococcal disseminated infection in an immunocompetent cat: Letter to the Editor, Vet. Dermatol. 22 (2011) 232–234. doi:10.1111/j.1365-3164.2010.00948.x.

[6] P.H.M. Cardoso, F. de A. Baroni, E.G. Silva, D.C. Nascimento, M. dos A. Martins, W. Szezs, C.R. Paula, Feline Nasal Granuloma Due to Cryptoccocus gattii Type VGII, Mycopathologia. 176 (2013) 303–307. doi:10.1007/s11046-013-9686-4.

[7] D.A. de Paula, A.B. de Almeida, F.S. da Cruz, F.H. Furlan, E.M. Colodel, V.R. Sousa, L. Nakazato, V. Dutra, Occurrence and molecular characterization of cryptococcosis in dogs and cats in Mato Grosso, Brazil, Pesqui. Veterinária Bras. 34 (2014) 167–172.

[8] P.Q. de Lima, F. P. de Oliveira, J. A. Marciano. Cryptococcosis in a cat - Case report. Rev Cient Med Vet. n. 30 (2018).

[9] A.C. Balda, J.C. Gonçalves, R.C. Menezes, A.C.F. de Souza, G.D. Cruz. Invasive cutaneous cryptococcosis of the nasal planum in a cat. Clin. Vet. 133 (2018) 26-31.
